# Landscape genomics predicts climate change‐related genetic offset for the widespread *Platycladus orientalis* (Cupressaceae)

**DOI:** 10.1111/eva.12891

**Published:** 2019-11-22

**Authors:** Kai‐Hua Jia, Wei Zhao, Paul Andrew Maier, Xian‐Ge Hu, Yuqing Jin, Shan‐Shan Zhou, Si‐Qian Jiao, Yousry A El‐Kassaby, Tongli Wang, Xiao‐Ru Wang, Jian‐Feng Mao

**Affiliations:** ^1^ Beijing Advanced Innovation Center for Tree Breeding by Molecular Design National Engineering Laboratory for Tree Breeding Key Laboratory of Genetics and Breeding in Forest Trees and Ornamental Plants Ministry of Education College of Biological Sciences and Technology Beijing Forestry University Beijing China; ^2^ Department of Biology San Diego State University San Diego CA USA; ^3^ Department of Forest and Conservation Sciences Faculty of Forestry The University of British Columbia Vancouver BC Canada; ^4^ Department of Ecology and Environmental Science UPSC Umeå University Umeå Sweden

**Keywords:** adaptation, climate change, genetic offset, genotyping by sequencing, *Platycladus orientalis*, population structure

## Abstract

Understanding and quantifying populations' adaptive genetic variation and their response to climate change are critical to reforestation's seed source selection, forest management decisions, and gene conservation. Landscape genomics combined with geographic and environmental information provide an opportunity to interrogate forest populations' genome‐wide variation for understanding the extent to which evolutionary forces shape past and contemporary populations' genetic structure, and identify those populations that may be most at risk under future climate change. Here, we used genotyping by sequencing to generate over 11,000 high‐quality variants from *Platycladus orientalis* range‐wide collection to evaluate its diversity and to predict genetic offset under future climate scenarios. *Platycladus orientalis* is a widespread conifer in China with significant ecological, timber, and medicinal values. We found population structure and evidences of isolation by environment, indicative of adaptation to local conditions. Gradient forest modeling identified temperature‐related variables as the most important environmental factors influencing genetic variation and predicted areas with higher risk under future climate change. This study provides an important reference for forest resource management and conservation for *P. orientalis*.

## 1 INTRODUCTION

The observed rapid pace of climate change is expected to profoundly influence species distribution and diversity, and is considered as one of the significant causes of biodiversity decline and/or loss in the next century (Dawson, Jackson, House, Prentice, & Mace, 2011; Pacifici et al., 2015; Warren et al., 2013). Evidence of climate‐induced local extinction is widespread among plant and animal species (Urban, 2015; Wiens, 2016). Forest trees constitute a significant group of organisms in their combined ecological and economic importance. Understanding how forest trees respond to climate change aids efforts to predict species range shift and informs management issues related to conservation and reforestation.

Long‐lived tree species with wide distribution ranges often show clear adaptation to local environments. Local adaptation in which local genotypes have a fitness advantage than foreign genotypes is well known among tree species (Aitken & Bemmels, 2016; Hereford, 2009). Rapid climate change can break this genetic–environmental association much faster than trees' ability to adapt in situ or migrate (Aitken, Yeaman, Holliday, Wang, & Curtis‐McLane, 2008; Jump & Penuelas, 2005), thus creating a mismatch between genetic adaptation to altered environmental conditions. In addition, human activities lead to population fragmentation, thereby reducing gene flow, which undoubtedly increases the risk of maladjustment of local populations when environment changes.

The development of landscape genomics is providing unprecedented insights into the evolutionary processes and the molecular basis of adaptation, aiding in understanding how species and populations respond to climate change challenges (McKinney, Larson, Seeb, & Seeb, 2017). Landscape genomics, integrating geographic and environmental information, uses a large number of genetic loci to understand the extent to which climate has shaped genetic variation in the past (Sork et al., 2013). It could further be used to quantify modern patterns of interaction between genetic variation and climate conditions, and predict vulnerable populations under future conditions when combined with methods for exploring nonlinear genotype–environment relationships in multivariate space (Fitzpatrick & Keller, 2015; Holliday et al., 2017). Gradient forest (GF) is a community‐level transfer function based on machine‐learning regression tree approach known as random forests (Ellis, Smith, & Pitcher, 2012). This method is now extended to analyze and map genomic variation associated with environmental tolerance across space and times (Fitzpatrick & Keller, 2015). GF modeling can also be used to calculate the difference between current and future genotype–environment relationships and forecast the geographic regions of high genetic mismatch under future climates if migration and de novo mutations cannot compensate for the required diversity. Until now, this approach has not been widely implemented in forest tree population studies.

*Platycladus orientalis* is a member of the family Cupressaceae and one of the dominant coniferous tree species in northern China. The natural range of *P. orientalis* covers northern and northwestern China, Korea, and Russian's Far East, and it is globally introduced to Africa, Asia, Australia, Europe, North America, and South America (Li, Du, & Wen, 2016). Due to the diverse geographic regions it occupies, the species exhibits large amounts of morphological and physiological variation (Mao et al., 2010; Shi, Zheng, & Qu, 1992; Wu, 1986). However, it is not clear whether these variations are reflective of genetic diversity and what evolutionary forces have driven the diversity.

In this study, we sampled the Chinese range of *P. orientalis* and surveyed their genetic variation using genotyping by sequencing (GBS). For large conifer genomes, GBS has become a practical method for generating genome‐wide variant data for population genetic studies (Chen, Mitchell, Elshire, Buckler, & El‐Kassaby, 2013; Pan et al., 2015; Parchman, Jahner, Uckele, Galland, & Eckert, 2018; Xia et al., 2018). Our objectives were (a) to assess the species' genetic diversity and population structure, (b) to evaluate the impact of environment and geography on genetic variation, and (c) to predict genetic offset of regional populations in relation to climate change. These investigations offer insight on environmental factors that have influenced the distribution of genetic diversity in this major conifer species, and provide basic information for forest managers to address management and conservation strategies under future climatic conditions.

## 2 MATERIALS AND METHODS

### 2.1 Sampling, GBS library preparation, and sequencing

**Figure 1:**
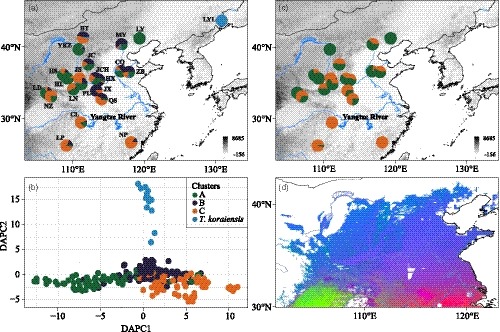
Population structure and gene–environment associations in *Platycladus orientalis*. (a) Pie chart shows the ancestral composition of each population with *K* = 4 inferred from ADMIXTURE. (b) DAPC of the 21 populations assigned to four clusters (a, b, c, and *Thuja koraiensis* as in panel a). (c) Genetic structure and admixture inferred with spatial conStruct (*K* = 2). (d) Gradient forest mapped genetic–environmental associations across the distribution area. Colors represent the PCA‐summarized gradients in genomic turnover. The first three PCs were each assigned to a RGB color, red, green, and blue. Similar colors in the sampled space correspond to similar expected genetic composition associated with climate

During the 1980s–1990s, bulked seeds from 21 *P. orientalis* populations distributed throughout China and one *Thuja koraiensis* population (LYL) from Heilongjiang Province, China, were collected (Table 1, Figure 1) and stored at –20℃ for further use. Populations from the south of the Yangtze River (CL, LP, NP) are in small, sporadically distributed patches and appear to be introduced (Dong, Chen, Zhang, Li, & Kong, 1990). *Thuja koraiensis* is morphologically similar to *P. orientalis* and has an adjacent distribution to the northeast of *P. orientalis*. We included *T. koraiensis* to test whether it can be distinguished by GBS and whether there is gene flow between these two species. From each population, 8–17 seeds were treated with 30% hydrogen peroxide for 1 hr and immersed in water overnight, and then germinated at 25℃ on moist filter paper (Table 1). DNA was extracted from seedlings using the cetyltrimethylammonium bromide (CTAB) method (Doyle & Doyle, 1987).

**Table 1:** Geographic locations, sample size (*N*), average heterozygosity per locus (*H*_obs_), average nucleotide diversity (*π*), and Wright's inbreeding coefficient (*F*_IS_) of the 21 *Platycladus orientalis* populations and one *Thuja koraiensis* population

Species	Cluster	Population	*N* (sampled)	*N* (valid)	Longitude (°E)	Latitude (°N)	*H*_obs_	*π*	*F*_IS_
*T. koraiensis*		Laoyeling (LYL)	17	13	131.04	43.55	0.22	0.0024	0.05
*P. orientalis*	A	Lingyuan (LY)	17	14	119.35	41.23	0.17	0.0025	0.18
	A	Yikezhao (YKZ)	15	13	110.80	39.60	0.20	0.0027	0.16
	A	Heshui (HS)	10	10	108.68	36.12	0.24	0.0028	0.09
	A	Huangling (HL)	11	11	109.27	35.58	0.26	0.0028	0.05
	A	Pinglu (PL)	11	9	111.22	34.84	0.22	0.0029	0.18
	A	Liangdang (LD)	15	14	106.30	33.58	0.21	0.0027	0.17
	B	Miyun (MY)	16	14	116.83	40.38	0.22	0.0026	0.10
	B	Jiaocheng (JC)	17	17	112.17	37.56	0.20	0.0028	0.20
	B	Jincheng (JCH)	17	15	113.12	35.58	0.24	0.0028	0.11
	B	Changqing (CQ)	14	14	116.73	36.60	0.22	0.0029	0.17
	B	Zibo (ZB)	17	17	117.85	36.50	0.24	0.0028	0.11
	B	Huixian (HX)	17	14	113.70	35.40	0.17	0.0023	0.14
	B	Jiaxian (JX)	10	10	113.30	33.90	0.22	0.0025	0.06
	C	Queshan (QS)	14	14	114.03	32.70	0.20	0.0026	0.15
	C	Luonan (LN)	15	12	110.07	34.10	0.20	0.0028	0.19
	C	Jishan (JS)	13	11	110.95	35.58	0.25	0.0027	0.05
	C	Nanzheng (NZ)	10	6	106.94	33.07	0.20	0.0025	0.09
	C	Cili (CL)	15	8	111.15	29.44	0.16	0.0021	0.09
	C	Liping (LP)	9	5	109.15	26.23	0.21	0.0024	0.04
	C	Nanping (NP)	12	9	118.17	26.65	0.19	0.0024	0.11
	Not defined	Baotou (BT)	8	4	111.42	41.33	0.22	0.0026	0.06

We prepared a GBS library for the 300 samples using a *Pst*I high‐fidelity restriction enzyme (New England Biolabs, MA, USA), following a previously established protocol (Pan et al., 2015) with minor modification. In brief, restriction enzyme digestion and adapter (with individual barcode) ligation were carried out simultaneously on 200 ng DNA from each sample. Then, the digested and ligated DNA were pooled, purified, and PCR‐amplified. Fragment size of 330–550 bp was selected and purified. Paired‐end sequencing (2 × 125 bp) was performed on an Illumina HiSeq2500.

### 2.2 Processing of Illumina data

Adapter sequences and low‐quality bases (base quality <20) from the tail of each read were removed using Trimmomatic v0.36 (Bolger, Lohse, & Usadel, 2014). Then, variants were built de novo from the short reads using Stacks pipeline (Catchen, Amores, Hohenlohe, Cresko, & Postlethwait, 2011). Briefly, the cleaned paired reads were demultiplexed and trimmed to 99 bp in length using the “process_radtags” module. The matching reads were grouped into stacks and built loci de novo in each sample with “ustacks” modules. The minimum number of reads to create a stack (‐m flag) was set at 3 following the strategy proposed recently (Paris, Stevens, & Catchen, 2017; Rochette & Catchen, 2017) with “‐H” flag to disable haplotypes calling from secondary reads, and the maximum distance (in nucleotides) allowed between stacks to define loci (‐M flag) was set at 4. After that, loci were matched up according to sequence similarity to create a catalog of all loci (i.e., a set of consensus loci) across the samples using “cstacks”; the distance allowed between sample loci (‐n flag) was set to 5. The number of polymorphic loci shared by 80% of samples was used to determine the values of parameters M and n for “ustacks” and “cstacks” modules (Figure S1; Paris et al., 2017; Rochette & Catchen, 2017). The settings of these parameters were used to control the number of SNPs recovered, measures of genetic diversity estimates, and genetic inference for downstream applications (Mastretta‐Yanes et al., 2015; Shafer et al., 2017). Then, the “sstacks,” “tsv2bam,” “gstacks,” and “populations” modules were implemented with default parameters to match each sample against the catalog and perform variants calling.

Subsequently, the variant dataset was further filtered using the “populations” module in Stacks and VCFtools (v0.1.13) (Danecek et al., 2011). Potential homeology was excluded by removing markers showing heterozygosity >0.70. SNPs with more than 50% of missing data were removed. We further filtered the dataset with a minor allele frequency (MAF) <0.05 and only kept biallelic SNPs.

### 2.3 Population genetic analyses

The population structure was investigated using the model‐based evolutionary clustering approaches as implemented in ADMIXTURE v1.30 (Alexander & Lange, 2011; Alexander, Novembre, & Lange, 2009) and discriminant analysis of principal components (DAPC) in R package adegenet (Jombart, 2008). Only one SNP from each GBS fragment was kept in ADMIXTURE analysis (3,911 SNPs). ADMIXTURE was run under *K* ranged from 1 to 10 and was repeated 10 times for each *K* with different random seeds. The most appropriate *K* value was selected after performing the 10‐fold cross‐validation procedure, whereby the best *K* exhibits low cross‐validation error (CV error) opposed to others. We used the CLUMPAK (Cluster Markov Packager Across *K*) web server to align and visualize the bar graphs (Kopelman, Mayzel, Jakobsson, Rosenberg, & Mayrose, 2015). DAPC with prior clusters defined by ADMIXTURE was carried out using the same set of SNPs.

To avoid overestimating the number of potential clusters caused by the presence of isolation by distance (IBD), as is often found in continuous populations, we further used conStruct (Bradburd, Coop, & Ralph, 2018) to identify structure in a spatially aware context. conStruct allows for explicit test of discrete versus continuous spatial patterns by estimating the ancestral components of each population and the rate at which relatedness decays with distance. We tested both spatial and non‐spatial models using loci of missing rate <30% (1,546 SNPs) for a range of *K* = 1–6, with 10 repetitions per each *K* value and 50,000 iterations per repetition. We performed 10‐fold cross‐validation to choose the best‐fit number of clusters (*K*). For each best fit *K*, we conducted three independent runs to evaluate the convergence.

Population differentiation (*F*_ST_) (Weir & Cockerham, 1984) between clusters was calculated using the R package hierfstat (Goudet, 2005). Population genetic statistics, including nucleotide diversity per base pair (*π*), Wright's inbreeding coefficient (*F*_IS_), and observed heterozygosity (*H*_obs_), were calculated using the “populations” module in Stacks.

### 2.4 Environmental variables and their associations with genetic variation

For each sampling location, we used a high‐resolution climate database, climateAP (Wang, Wang, Innes, Seely, & Chen, 2017), to generate environmental data for China. We chose 49 variables with known impacts on plant survival and development, including 14 annual and 35 seasonal environmental variables (Table S1). We calibrated and downscaled climatic projections representing two different future scenarios RCP4.5 and RCP8.5, reflecting moderate and extreme conditions, respectively, for 2055 and 2085.

We performed GF analyses to identify the environmental variables that best explained the distribution of genetic diversity using the R package gradientForest (Ellis et al., 2012). To ameliorate some of the problems that arise due to linkage among markers, we kept only one SNP per GBS fragment for GF analyses. Any SNP that was polymorphic in fewer than 5 of the 17 populations was removed to ensure attaining robust regression. We used 2,000 regression trees per SNP to fit GF model while keeping all the parameters set at default values.

**Figure 2:**
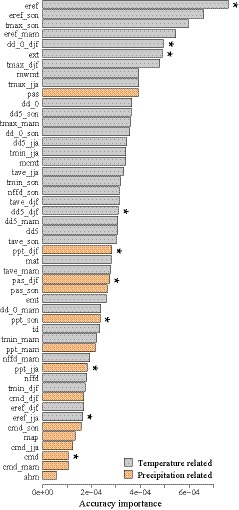
Environmental variables used in the gradient forest modeling. Variables were ordered by ranked importance. *Top‐ranked, uncorrelated environment variables (Pearson's |*r*| < .8)

At first, all the 49 environmental variables were included in GF models. After evaluating the ranked importance and Pearson pairwise correlations among these variables, 10 variables (eref, dd_0_djf, ext, dd5_djf, ppt_djf, pas_djf, ppt_son, ppt_jja, eref_jja, and cmd) with absolute value of Pearson correlation coefficient (*r*) ≤.8 were retained for the following analysis (Figure 2; Table S1). The 10 environmental variables and the unlinked SNPs were used to build the final GF model, which was used to predict the current genomic composition of each grid point across the range of *P. orientalis* as defined by Hu, Jin, Wang, Mao, and Li (2015). The resulting multidimensional genomic patterns were summarized using principal component analysis (PCA) (Ellis et al., 2012). The first three PCs were each assigned to a RGB color, red, green, and blue. Similar colors in the sampled space correspond to similar expected genetic composition. This allows us to visualize the different environmental adaptations within the distribution of *P. orientalis*, where similar colors represent similar allele frequencies.

### 2.5 Isolation by distance (IBD) and isolation by environment (IBE)

To investigate the role of geographic and environmental factors in shaping the spatial genetic differentiation, we calculated: (a) isolation by distance (IBD), (b) isolation by environment (IBE), and (c) the correlation between environmental and geographic distance. Pairwise *F*_ST_/(1 − *F*_ST_) (Rousset, 1997) was calculated among populations using the R package hierfstat (Goudet, 2005). Mantel test was used to assess associations between linearized *F*_ST_/(1 − *F*_ST_) and geographic distance and environmental distance with significance determined using 999 permutations in the R package vegan (Oksanen et al., 2018). The 10 selected climate variables were used to calculate environmental distances by first scaling and centering the variables to account for differences in magnitude, then calculating pairwise Euclidean differences between sites.

### 2.6 Outlier detection

We applied three methods to detect outlier SNPs, BayeScan (Beaumont & Balding, 2004), Pcadapt (Luu, Bazin, & Blum, 2017), and Bayenv2 (Günther & Coop, 2013). For BayeScan, we designated 30 prior odds for the neutral model and included 100 pilot runs, followed by 100,000 iterations with a burn‐in length of 50,000 iterations (Lotterhos & Whitlock, 2014). To decrease false positives due to multiple testing, we applied the false discovery rate (FDR) criterion (0.01). For Pcadapt, the *K* = 3 was selected based on scree plot (Figure S2). Outlier SNPs were identified under FDR of 0.01 using *q*‐value package in R (Storey, Bass, Dabney, & Robinson, 2015). For Bayenv2, we first created a neutral SNP set by excluding any outlier SNPs detected by BayeScan and Pcadapt, and retaining one SNP per GBS fragment. The covariance matrix was estimated using these SNPs with 100,000 iterations. We compared three independent runs of covariance matrices with different random seeds to ensure convergence. The same 10 environmental variables were used to calculated environmental correlations by averaging five independent runs of Bayenv2 with 100,000 Markov chain Monte Carlo (MCMC) iterations with different random seeds. We considered the SNPs in the top 1% of Bayes factor (BF) values (BF > 3) and top 5% of the absolute value of Spearman rank correlation coefficients (*ρ*) as significant putative adaptive loci.

### 2.7 Redundancy analysis

To estimate the degree to which genomic variation is influenced by environmental or geographic variables, we performed a series of redundancy analyses (RDAs) in the R package vegan (Oksanen et al., 2018). RDA involves a multiple linear regression followed by a PCA on the matrix of regression‐fitted values. A dependent matrix of minor allele frequencies for each population, and two independent matrices of environmental variables and geographic variables were included. For the geographic matrix, we used Moran's eigenvector map (MEM) to calculate the spatial weighted matrix of the sampling sites using the R package adespatial (Dray et al., 2018). Only the top three eigenfunctions (MEM1, MEM2, and MEM3) representing significant positive spatial correlation were retained in the RDA following the recommendation of Borcard, Gillet, and Legendre (2011). For environmental matrix, forward selection was used to reduce the number of variables in the model with a stringent alpha value of 0.05. After that, to further avoid high collinearity, we excluded those with a variance inflation factor (VIF) over 10 (Borcard et al., 2011). Finally, we reserved two environmental variables, including Hargreaves reference evaporation (eref) and degree‐days above 5°C in December, January, and February (dd5_djf) to explain population variation using the rda function in the vegan package (Oksanen et al., 2018). The ANOVA.cca function was used to test the significance of the partitioning with 999 permutations.

### 2.8 Genetic offset under future climates

To identify the spatial regions where genetic–environmental relationships will be most likely disrupted by climate change, we first used the current GF model to predict genetic compositions under RCP4.5 and RCP8.5 for the years 2055 and 2085. Then, we calculated the Euclidean distances between the current and predicted future genomic composition to represent the genetic offset between current and future climates across the landscape (Bay et al., 2018; Fitzpatrick & Keller, 2015). We visualized the genetic offset for different climate scenarios in geographic space to show the spatial distribution of population‐level vulnerability to climate change.

## 3 RESULTS

### 3.1 Sequence data

Our GBS generated 520 million paired‐end reads from 300 individuals, of which 472 million reads (90.7%) passed initial quality filters (Table S2). A total of 46 individuals with low coverage (<0.1 million reads) were discarded, leaving 254 valid samples in this study, with 4–17 individuals per population across the 22 sampled populations (Table 1). Under our parameter settings, Stacks initially recovered 704,684 SNPs, and after filtering for MAF (≥0.05), missing rate (≤0.5), and heterozygosity (≤0.7), the number of SNPs was reduced to 11,049. We further created a set of unlinked SNPs by keeping only one SNP per GBS fragment; this set consisted of 3,911 SNPs.

### 3.2 Population genetic structure

Using the unlinked SNPs (3,911), ADMIXTURE identified *K* = 4 as the most likely number of evolutionary clusters among the 22 populations sampled, including one population of *T. koraiensis* (Figure S3A). Under *K* = 3, the 22 populations were divided into a *T. koraiensis* cluster (LYL), a cluster A with 6 populations (LY, YKZ, HS, HL, PL, and LD), and a large third cluster with all the remaining populations (Figure S3B). Under *K* = 4, the *T. koraiensis* and the A clusters were maintained unchanged, but the third cluster was split into B (MY, JC, JCH, CQ, ZB, HX, JX) and C (QS, LN, JS, NZ, CL, LP, NP) clusters (Figure 1a; Figure S3B). One population (BT) was removed from further analyses due to its small valid sample size (*n* = 4) and being a likely introduced population. DAPC showed clear separation among the clusters identified by ADMIXTURE (Figure 1b). The first discriminant axis (DAPC 1) revealed a separation of *P. orientalis* populations, while the second (DAPC 2) highlighted the divergence between *T. koraiensis* and *P. orientalis*.

conStruct cross‐validations showed that the spatial model was marginally superior to non‐spatial model as the predictive accuracy of non‐spatial model continued to improve slightly as subsequent clusters were added up to *K* = 6 (Figure S4), indicative of overestimating the number of potential clusters. For the spatial model, the predictive accuracy was highest at *K* = 3, but additional spatial layer beyond *K* = 2 contributed very little to total covariance (Figure S4). Thus, the spatial model at *K* = 2 sufficiently described the population structure, and the clustering patterns of spatial and non‐spatial models were very similar, indicating the contribution of IBD to the population structure was small. The decay of genetic relatedness against the geographic distance also supported weak IBD within each ancestral layer (α_D_ ≈ 0) (Figure S5). The pattern of admixture along the latitudinal gradient revealed by conStruct was similar to the results of ADMIXTURE (Figure 1a,c; Figure S4).

Genetic differentiation (*F*_ST_) among clusters (A, B, C, and *T. koraiensis*) was significant when using unlinked 3,911 SNPs, with *F*_ST_ values ranged from 0.030 to 0.140 (Table S3). The differentiation between *T. koraiensis* and *P. orientalis* (*F*_ST_ = 0.105–0.140) was noticeably higher than within *P. orientalis* (0.030–0.069). On all 11,049 SNPs, *F*_ST_ values changed little (Table S3). The average genetic diversity (*π*) in *P. orientalis* populations ranged from 0.0021 to 0.0029 when considering all 11,049 SNPs, and the value in *T. koraiensis* was similar (0.0024, Table 1).

### 3.3 Environmental associations with genetic variation

Three small, sporadically distributed, and potentially introduced *P. orientalis* populations located south of the Yangtze River (CL, LP, NP) and the *T. koraiensis* population (LYL) were removed from this analysis, leaving 17 populations for all following analyses. We performed GF analyses to test whether a subset of genomic variation can be explained by environmental effects and to visualize climate‐associated genetic variation across the species range. Mapping of genetic variation across environmental space revealed significant differences of genetic composition along the latitudinal and longitudinal axes of *P. orientalis* range (Figure 1d). GF identified Hargreaves reference evaporation (eref) as the most important predictor among environmental variables considered, followed by autumn Hargreaves reference evaporation (eref_son) and autumn mean maximum temperature (tmax_son) (Figure 2). The top nine environmental factors (eref, eref_son, tmax_son, eref_mam, dd_0_djf, ext, tmax_djf, mwmt, and tmax_jja) were all related to temperature, suggesting temperature was a key factor influencing distribution of *P. orientalis* (Figure 2).

### 3.4 Partitioning genomic variation to IBD and IBE

**Figure 3:**
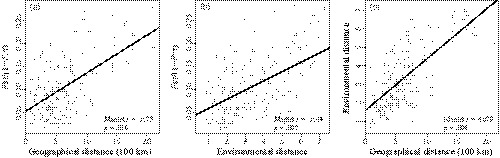
Isolation by distance and environment using Mantel test. (a) Pairwise genetic distance *F*_ST_/(1 − *F*_ST_) is significantly associated with geographic distance and (b) environmental distance. (c) Geographic distance is significantly correlated with environmental distance

Gene flow patterns may align with environmental or geographic distances, so we tested isolation by distance and environment. A significant correlation between pairwise *F*_ST_(1 − *F*_ST_) and Euclidean geographic distance (Mantel *r* = .3573, *p* = .016; Figure 3a) was detected by the Mantel test, indicating a significant pattern of isolation by distance (IBD). We also identified a significant pattern of isolation by environment (IBE) based on distance derived from environmental deviation (Mantel *r* = .3544, *p* = .009; Figure 3b), and the level of correlation was similar to IBD. However, the autocorrelation between environmental and geographic distances was also strong (Mantel *r* = .4079, *p* = .004; Figure 3c). We therefore applied RDA to dissect the individual roles of IBD and IBE and their confounding effect on genomic differentiation.

From the full set of SNPs (11,049), we detected 579 (5.24%) outlier SNPs by the three detection methods used. BayeScan, Pcadapt, and Bayenv2 identified 214 (1.94%), 211 (1.91%), and 228 (2.06%) significant outlier SNPs, respectively (Figure S6). We performed RDA on outlier SNPs and the full set of SNPs. RDA results showed that environmental and geographic variables explained small but significant proportions of genetic differentiation on the 11,049 SNPs, as measured by adjusted *R* ^2^ (6.3%–10.7%, *p* < .05; “combined fractions,” Table 2). To further unlock the contribution between geography and environment, we performed partial RDA. A total of 13.1% of variation could be jointly explained by environment and geography (“total explained,” Table 2). Environment and geography each exclusively explained 6.8% (*p* < .05) and 2.4% (*p* > .05) of the variation, respectively.

**Table 2:** Redundancy analyses (RDAs) to partition among‐population genetic variation in *Platycladus orientalis* into environment (env), geography (geo), and their combined effects, shown in the table as measured by adjusted *R* ^2^

	All SNPs (11,049 SNPs)	Outliers		
		Bayenv2 (228 SNPs)	Pcadapt (211 SNPs)	BayeScan (214 SNPs)
Combined fractions				
F ~ env.	0.107**	0.158**	0.053^ns^	0.002^ns^
F ~ geo.	0.063**	0.111**	0.068^ns^	0.022^ns^
Individual fractions				
F ~ env.|geo.	0.068*	0.081*	0.005^ns^	−0.002^ns^
F ~ geo.|env.	0.024^ns^	0.036^ns^	0.020^ns^	0.018^ns^
F ~ env. + geo.	0.039	0.077	0.048	0.004
Total explained	0.131	0.193	0.073	0.020
Total unexplained	0.869	0.807	0.927	0.980
Total	1.000	1.000	1.000	1.000

Note

F: Dependent matrix of population allele frequencies; RDA tests are in the form of F ~ independent matrices|covariate matrixes. env.: environment (two variables); geo.: geography (three MEM variables). Total explained: total adjusted *R* ^2^ of individual fractions. Significance of confounded fractions (env. + geo.) between environment and geography was not tested.

Abbreviation: ns, not significant.

* *p* < .05; **p < .01.

Outlier SNPs may be constituted by environment or geography or both. We found 2%–19.3% of the variation could be explained by both factors jointly (Table 2; total explained). Variations in the Bayenv2 outliers showed bigger impact of environments than in the Pcadapt and BayeScan outliers. In the corrected RDA, environment exclusively explained 8.1% (*p* < .05) of the variation in the Bayenv2 outlier SNPs, but the effect of geography was insignificant (3.6%; *p* > .05), while the corresponding values were −0.2% to 0.5% (*p* > .05) and 1.8%–2.0% (*p* > .05) for the Pcadapt and BayeScan outliers (Table 2).

### 3.5 Genetic offset to climate change

**Figure 4:**
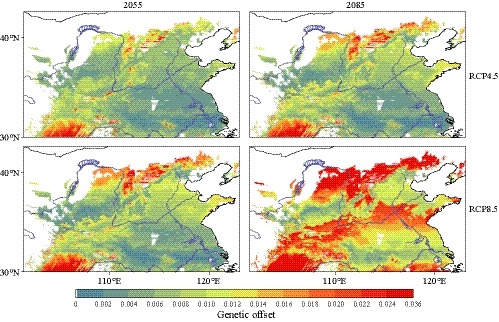
Predicted genetic offset across *Platycladus orientalis* distribution in the years 2055 and 2085 under scenario RCP4.5 and RCP8.5. Red and blue represent high and low genetic offset, respectively

GF modeling predicted regions with high genetic offset in the southern and northern margins of the *P. orientalis* distribution under future climate, with the southern range showing higher genetic offset, especially in the northeastern Sichuan Province (bounded by 103°E–108°E, 30°N–32°N; Figure 4) under RCP4.5 by 2055. As expected, under more severe climate change (RCP8.5) and longer time perspective (2085), the range and degree of genetic offset increased (Figure 4).

## 4 DISCUSSION

This population genomics study confirmed the genetic divergence between *P. orientalis* and *T*. *koraiensis*, identified population structure in *P. orientalis*, and revealed evidence of adaptive genetic variation through a combined *F*_ST_ outlier, genetic–environmental association, and RDA approach. Based on current genetic–environmental associations and machine‐learning‐based modeling, we identified regions with high genetic offset in *P. orientalis* distribution range where genetic–environmental relationships are most likely to be disrupted under future climate conditions. Our analyses provide the first insight on diversity and evolutionary forces operating in this species and assist genetic conservation and reforestation operations.

### 4.1 Population structure

Strong genetic differentiation between *P. orientalis* and *T*. *koraiensis* populations supports their divergence as two different species. The taxonomic status of the two species is debated, as *P. orientalis* is also named as *T. orientalis* (Durak et al., 2019; Xie, Dancik, & Yeh, 1992). The intermediate positions of some individuals of *T*. *koraiensis* suggest a probability of gene exchange between the two species. Mountains running through northeastern China, such as Greater Khingan, Lesser Khingan, and Baekdu Mountain, could provide barriers limiting pollen flow and form geographic isolation between these two species. Further validation of reproductive barrier between them would require crossing experiment and genetic testing.

Within *P. orientalis*, ADMIXTURE and conStruct indicated 2–3 genetic clusters with prominent admixture across regions. The spatial clustering identified by ADMIXTURE and conStruct was largely congruent with each other showing a clear south–north transition (Figure 1). conStruct gave little support to an overall pattern of IBD in the sampled populations, as also shown by geographically adjacent populations were not always more similar than geographically distant population, such as LD and NZ, MY, and LY. These results suggest that some populations of *P. orientalis* may have been in isolation in the past, likely resulted from historical processes (i.e., geographic isolation or refugia). Geological events and subsequent climatic changes during Pliocene–Pleistocene in northern and western China were identified as major forces that have shaped the distribution and genetic differentiation of forest species in northern China (Xia et al., 2018). In addition, human activities also contributed to the fragmentation of *P. orientalis*. It is suggested that *P. orientalis* became fragmented into relatively small and isolated populations since at least 1,500 years ago (Xie et al., 1992). A more thorough sampling covering all isolated populations would provide a more detailed demographic history of the species.

### 4.2 Environmental adaptation

Environment has been widely reported as a strong selective pressure on natural populations (Joshi et al., 2001; Mosca et al., 2012). Testing for IBD and IBE in *P. orientalis* using Mantel test indicated that geographic and environmental distances were almost equally important to the observed genetic differences. The relationship between geographic and environmental distances was highly correlated, which made it difficult to correctly parse out the factor that plays the key role in shaping the genetic variation. We thus further applied RDA to subdivide the genetic variation to environment and geography. On all SNPs, 13.1% of the variation could be jointly explained by the environment and geography, with environment exclusively contributing 6.8%, leaving 86.9% of the genetic variation unexplained. For the outlier SNPs, especially the Bayenv2 outliers, 19.3% of the variation could be explained by both environment and geography, of which a significant 8.1% of variation was attributed to environment, while geography was insignificant. These results suggest that environment was more important than geography in the population differentiation of *P. orientalis*. The genomic signature of IBE in *P. orientalis* was similar to that of *Pinus tabuliformis* in northern China (Xia et al., 2018). This signature of IBE can be produced by genetic adaptation to local environments (Nachman & Payseur, 2012; Wang & Bradburd, 2014).

Local adaptation studies on climate change contribute to understanding the ability of populations to sustain or adapt to rapid climate change (Fournier‐Level et al., 2011). Adaptive variation is partially structured by environmental factors, which may be mostly driven by temperature gradients for *P. orientalis* (Fu & Shen, 1989). GF analyses indicated that temperature was by far the most important variable associated with genetic variation. Temperature is a key factor influencing growth and phenology of various tree species, including *P. orientalis* (Fu & Shen, 1989). Temperature influences the growth of plants by affecting the metabolic processes such as photosynthesis, respiration, and transpiration, as well as the metabolic processes that affect the synthesis and transportation of organic matter (Wahid, Gelani, Ashraf, & Foolad, 2007). Additionally, ambient temperature can directly affect soil temperature, thus affecting the absorption and transport of water and nutrients. Low temperature (dd_0_djf) seemed to be an indispensable factor, which is not only a limiting factor for the survival (Dong et al., 1990) but also a critical factor for volume growth in *P. orientalis* (Chen, Yang, Li, Xu, & Wang, 2012). However, the physiological mechanism of *P. orientalis* responding to low temperature is not yet understood. Dissection of this adaptive mechanism should be the objective of future studies.

Water availability is commonly recognized as another critical factor delimitating tree species' distribution in northern China (Mao & Wang, 2011; Xia et al., 2018). However, for *P*. *orientalis* only one of the top ten‐ranked environmental variables is in the water regime indicating that precipitation has less impact on genetic adaptation than temperature. This is likely due to the strong drought tolerance of *P*. *orientalis*, as it can survive under annual precipitation of less than 200 mm and soil moisture content below 5% (Dong et al., 1990). Furthermore, seasonal precipitation (ppt_jja, ppt_son, ppt_man, ppt_djf) appeared to be more important than annual mean precipitation (map, Figure 2), suggesting that the time of precipitation might have a greater impact on phenological and periodic events such as flowering and growth than annual variables.

### 4.3 Genetic offset in future climates

Based on the current genetic–environmental associations, we attempted to assess the potential genetic offset in *P. orientalis* under future conditions using GF modeling. The same strategy has been applied to a variety of species (Bay et al., 2018; Fitzpatrick & Keller, 2015; Maier, 2018). Using this method, we gain insight into the potential risk of a species' persistence under climate change.

Our GF analyses suggested that *P*. *orientalis* would be less affected in the northwestern Loess Plateau and most of northern China, while relatively high genetic offset was predicted in the northern and southern margins. These high‐risk regions would need to adapt fast, either actively or passively, to keep pace with climate change; otherwise, populations in these regions may decline. Similar to other conifers, *P. orientalis* has a relatively long lifecycle with long generation intervals, in which the rate of emergence and spread of novel adaptive alleles in populations through de novo mutations are likely to be too slow to respond to rapid future climate changes.

The prediction accuracy of the GF models has been verified on bird populations (Bay et al., 2018; Ruegg et al., 2018). However, true evolutionary responses in long‐lived conifers are more complex to predict than GF models and may involve interactions between selection and the distribution of fitness effects of minor alleles and new mutations. Minor alleles were excluded from many analyses in this study due to consideration of genotyping errors in GBS procedure, for which we applied stringent filtering including MAF. Rare alleles may contribute to adaptation, and their roles should be better investigated using other genotyping methods (e.g., resequencing). Additionally, effective population size, the level of standing genetic variation, and the stage of population equilibrium in terms of local adaptation can influence the accuracy and power of GF projection and interpretation. Future work is needed to combine landscape genomics and empirical data on phenotypic variation of the *P. orientalis* populations to validate and adjust model predictions.

### 4.4 Management strategy

*Platycladus orientalis* is one of the most widely distributed coniferous trees in China (Dong et al., 1990) with a long life span, strong adaptability, and wide utilization. It is of great significance for accelerating the greening of China and improving the ecological environment. Due to the drought resistance, this species plays an important role in China's landscape, especially in the northwestern Loess Plateau and the establishment of protection shelterbelts in northern China (Dong et al., 1990). In the present study, we identified the genetic structure and differentiation within *P. orientalis* range, which offers an opportunity for optimal seed zone delineation, allocation of seed sources, seed movement for reforestation, and genetic conservation. For example, traditional seed transfer guidelines are based on provenance trials and climate models to select the range of seed transplants. However, establishment and maintenance of provenance trials are expensive and time‐consuming, resulting in limited information available, which makes it challenging to develop either population response functions or transfer functions (Mátyás, 1994). The knowledge generated from the present study could serve as complementary or an alternative to traditional approaches.

Considering that future climate may dramatically change in certain parts of the species' range, we propose adopting assisted gene flow to increase genetic diversity and adaptation to the anticipated climate changes. Assisted gene flow is a managed migration of individuals or gametes between populations within species range, which may be effective in accelerating adaptation to future climate (Aitken & Whitlock, 2013). In the areas of predicted high genetic offset, we should consider the use of a composite provenance that mixes native with selected non‐native seedlots to increase diversity and resilience. The main genetic clusters detected in our study were broadly distributed, encompassing large variations in growth, suggesting phenotypic variations evolve at a different pace. Thus, composite seed sourcing would allow faster climate matching without risking potential genetic mismatch. For example, in the north margin where the predicted genetic offset is high, it may be appropriate to introduce seeds from the warmer southern regions of the same climate zone, where the seeds may include pre‐adapted genotypes to future climate. It must be noted that our prediction is based on simulation of genomic information, without considering the function of other potential factors such as phenotypic plasticity and the stage of population equilibrium in terms of local adaptation. It would be valuable to conduct experiments on seedlings from different regions to be exposed to varying combinations of water and temperature to evaluate responses to environmental conditions. Such testing will help to refine seeds transfer strategy and validate genetic–environmental interactions.

## CONFLICT OF INTEREST

The authors declare no conflict of interest.

## AUTHOR CONTRIBUTIONS

JFM, XRW, TW, and YAE planned and designed the research; YJ, SSZ, SQJ, and WZ collected samples and executed the GBS prep; WZ and PAM contributed some scripts; KHJ, WZ, and XRW analyzed the data and wrote the manuscript; JFM, YAE, TW, WZ, and XRW edited and improved the manuscript; all authors approved the final manuscript.

## Data Availability

Sequencing reads have been deposited in the NCBI Sequence Read Archive (SRA) under BioProject PRJNA510176.
