# Supplementary material for: Landscape genomics predicts climate change‐related genetic offset for the widespread Platycladus orientalis (Cupressaceae)
Source: Evol Appl. 2019 Nov 22;13(4):665–76. doi: 10.1111/eva.12891 (PMC7086053; doi:10.1111/eva.12891)
Supplement: Supplementary file 1 [file EVA-13-665-s001.docx]

**
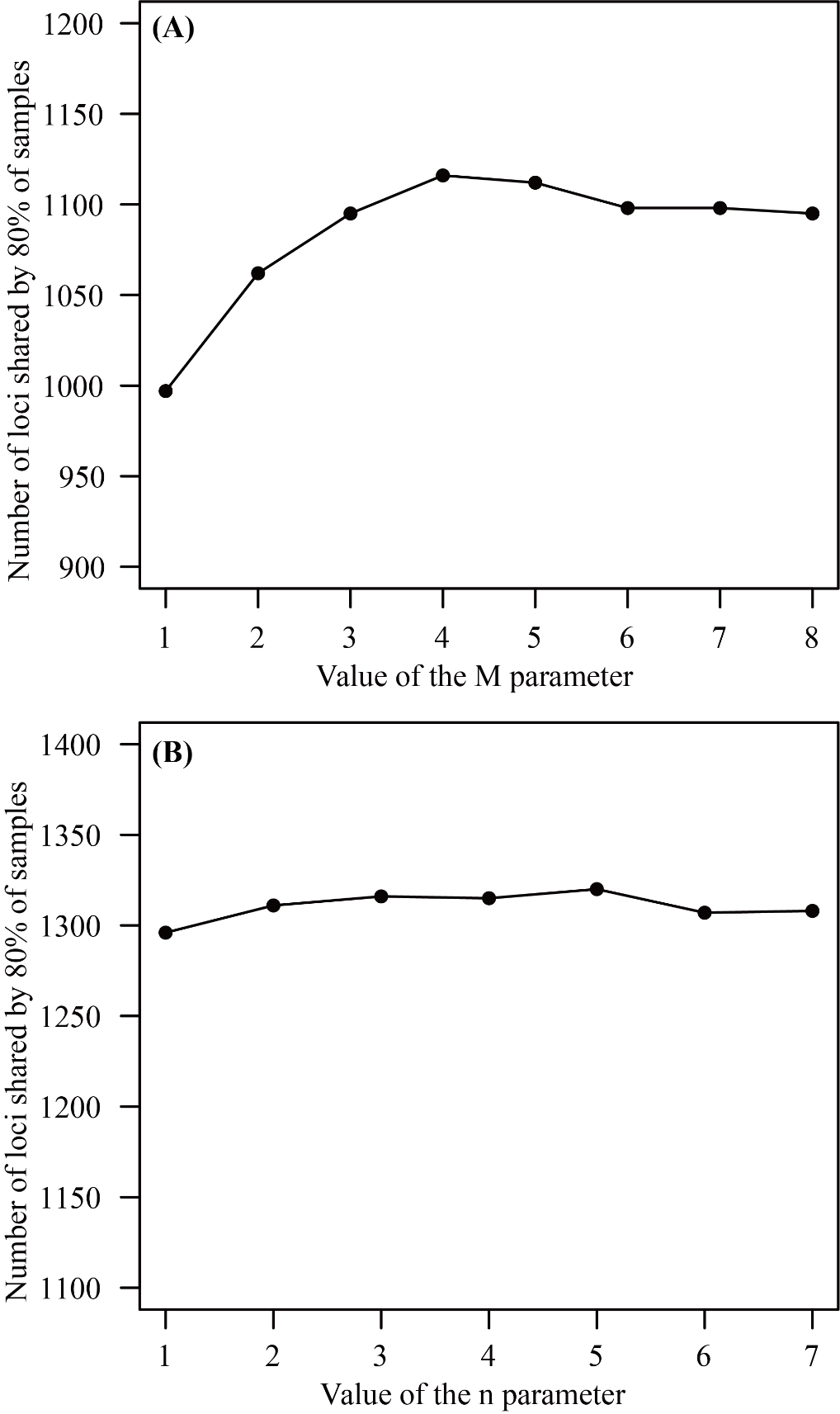
**

**Fig. S1.** Plots of the distribution of the number of loci shared by 80% of samples or more in different iterating values for (A) the maximum distance allowed between stacks to define a loci (M) and (B) the number of mismatches allowed between sample loci when build the catalog (n).


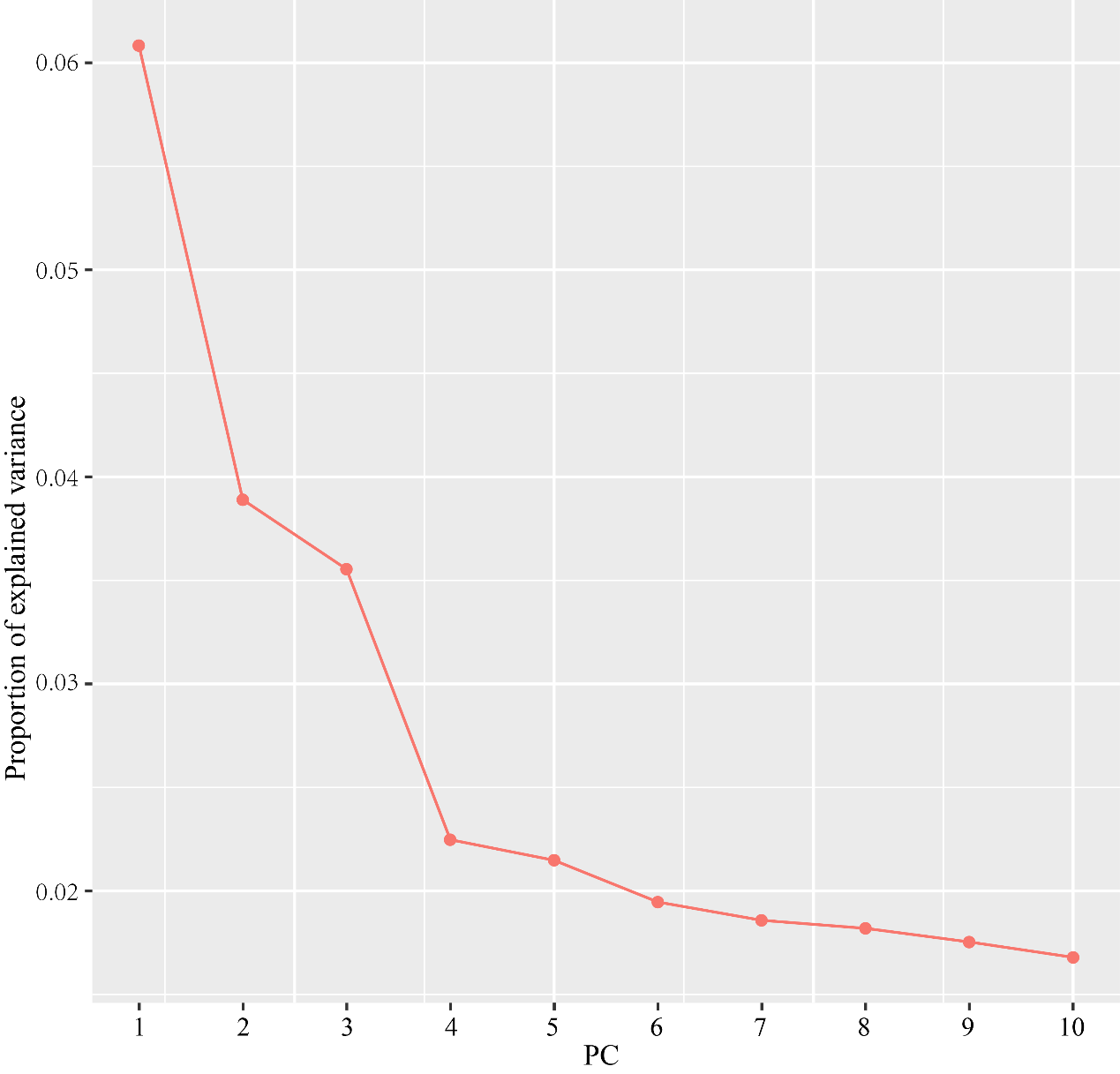


**Fig. S2.** Scree plot in Pcadapt displays the percentage of variance explained by each PC in a decreasing order.


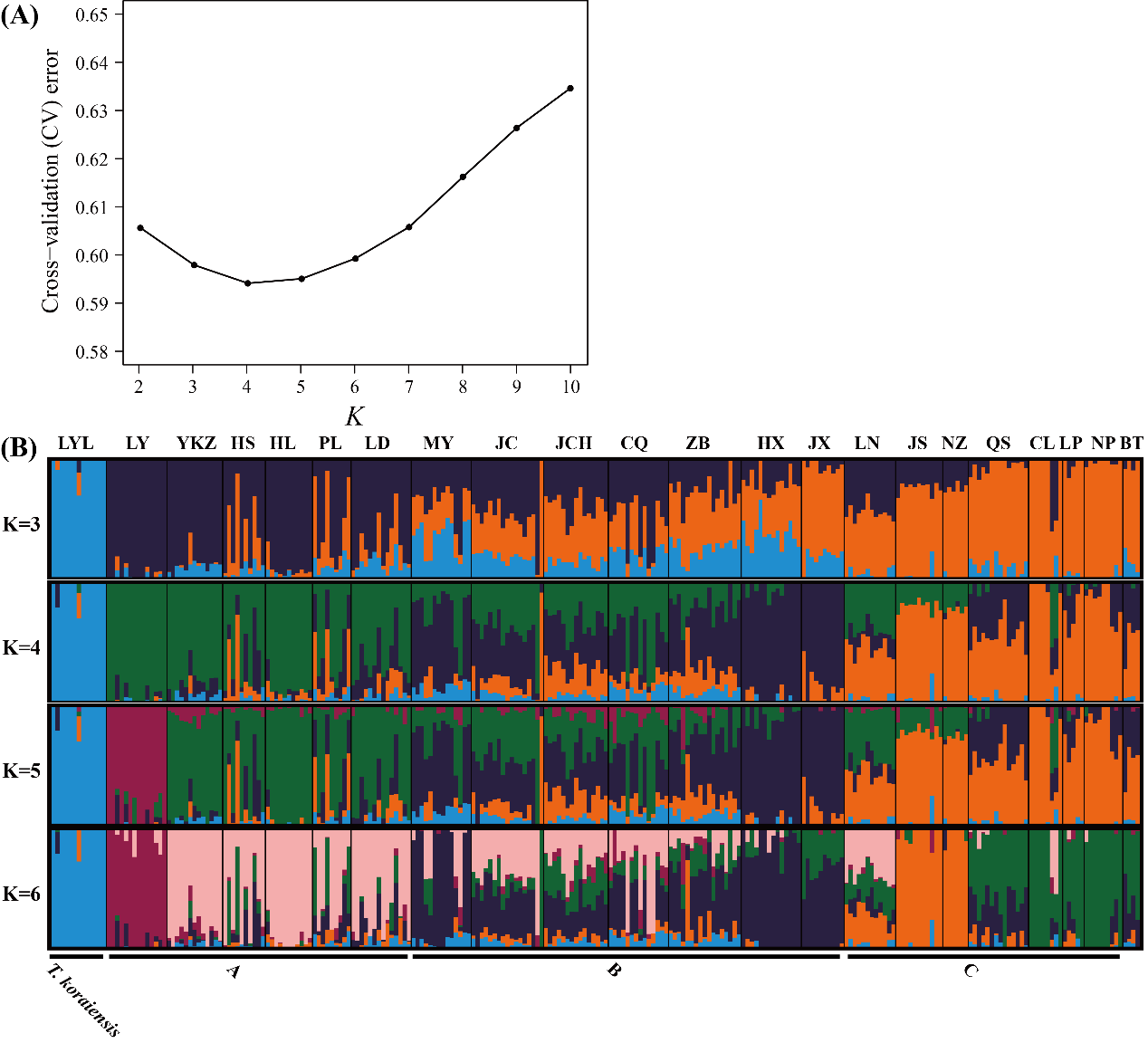


**Fig. S3.** ADMIXTURE analyses for *P. orientalis* and *T. koraiensis*. (A) Plot of ADMIXTURE cross-validation error. (B) Clustering of individuals (ADMIXTURE mode) for 21 *P. orientalis* populations and one *T. koraiensis* population with *K* = 3 – 6.

**
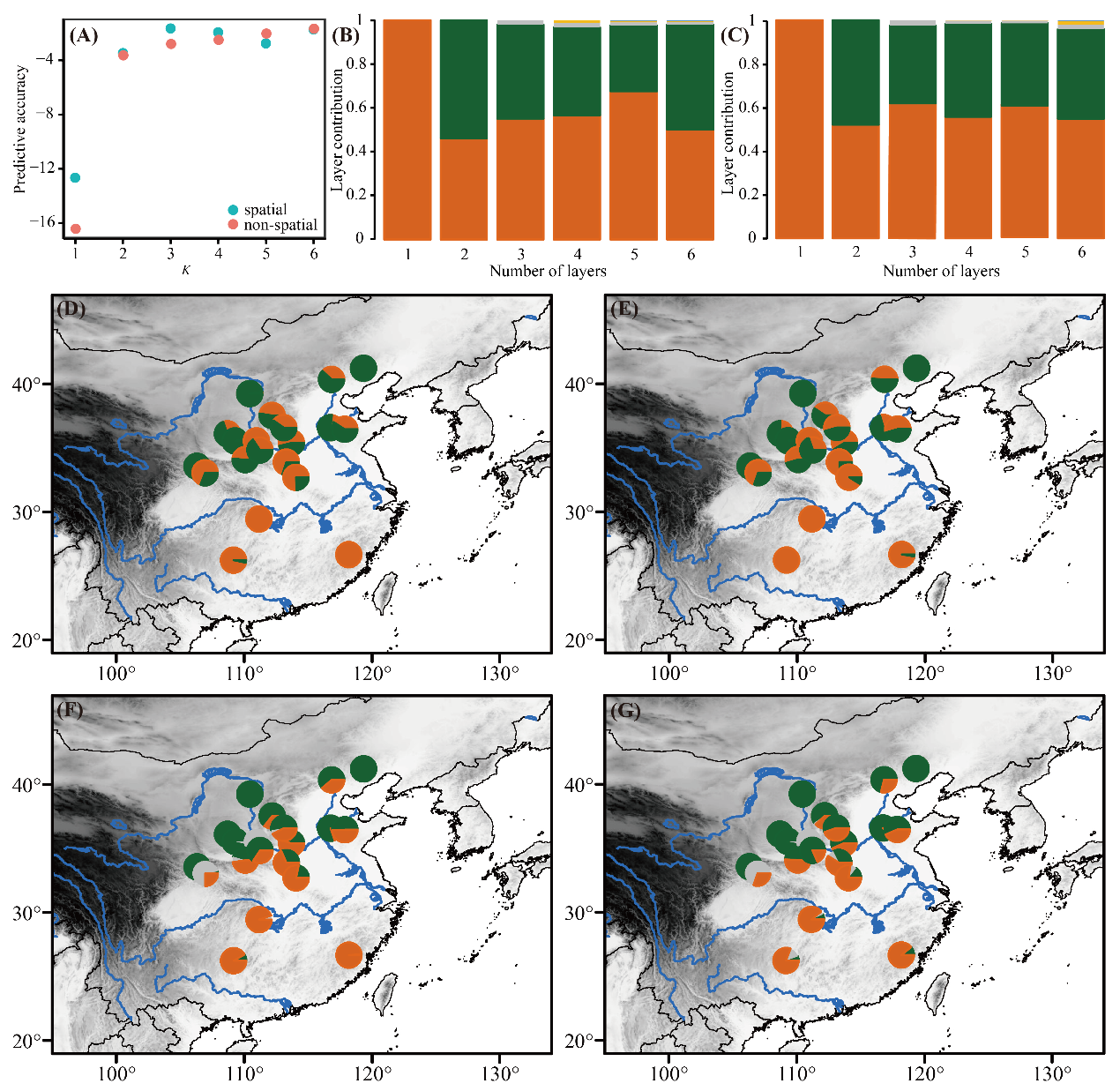
**

**Fig. S4.** Spatial population structure based on spatial and non-spatial conStruct models. (A) Cross-validation results comparing the spatial and non-spatial models with *K* = 1 – 6. (B) and (C) Ancestral layer contributions to the total covariance with *K* = 1 – 6 for the spatial and non-spatial model, respectively. (D) and (E) Population admixture estimated with *K* = 2 using spatial model and non-spatial model, respectively. (F) and (G) Population admixture estimated with *K* = 3 using spatial and non-spatial model, respectively.


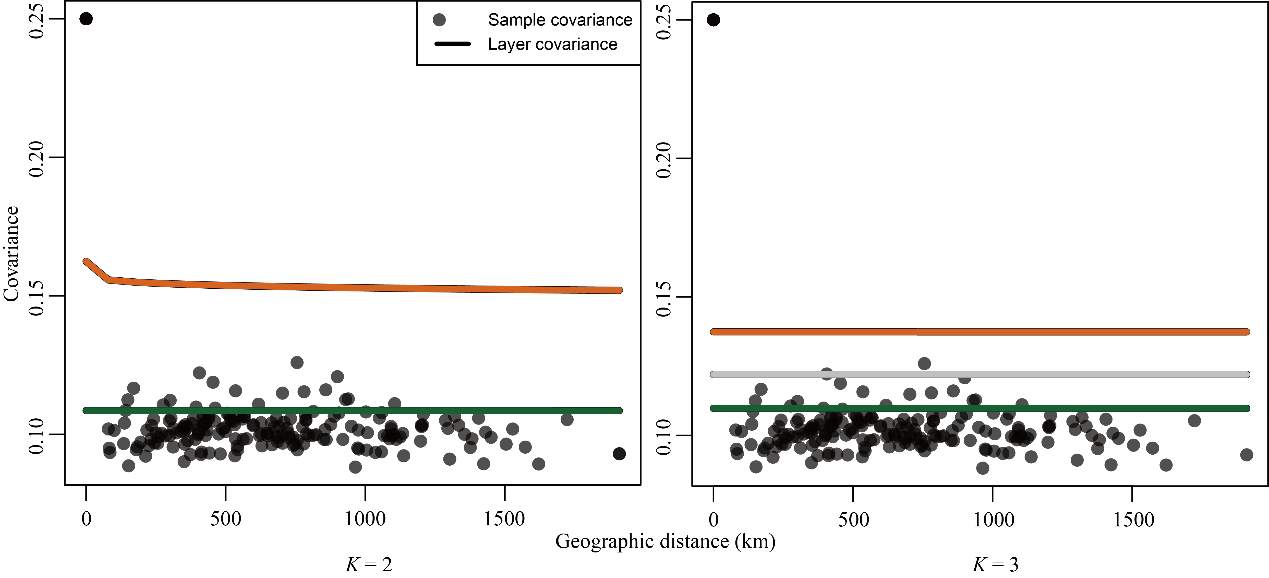


**Fig. S5.** Plots showing the layer-specific covariance curves using the spatial conStruct model for *K* = 2 (left) and *K* = 3 (right). Line colors are consistent with cluster colors in Fig. S4D, F.


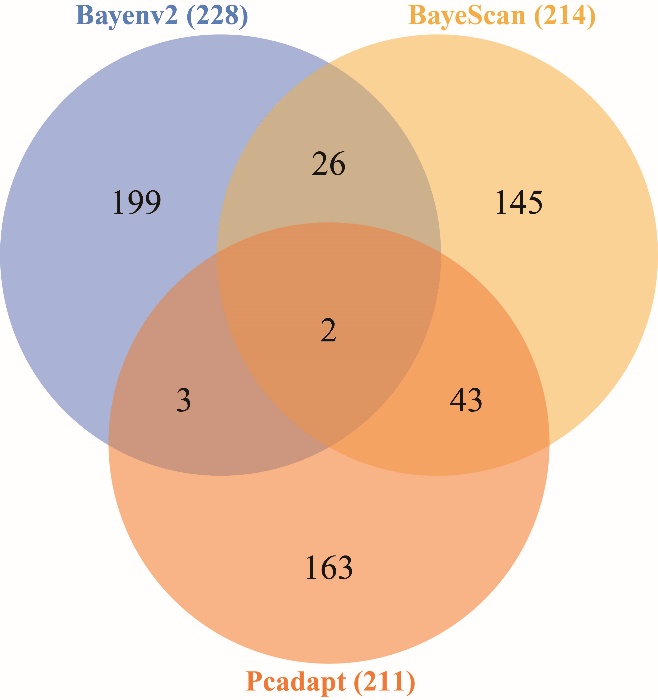


**Fig. S6.** No. of outlier SNPs identified by three different methods, Bayenv2, BayeScan, and Pcadapt.

**Table S1.** Environmental variables used in this study.

| **Categoriy** | **Code** | **Description** |
| --- | --- | --- |
| annual | ahm | annual heat: moisture index ((MAT+10)/(MAP/1000)) |
| annual | cmd | Hargreaves climatic moisture deficit |
| seasonal | cmd_djf | Hargreaves climatic moisture deficit in December, January and February |
| seasonal | cmd_jja | Hargreaves climatic moisture deficit in June, July and August |
| seasonal | cmd_mam | Hargreaves climatic moisture deficit in March, April and May |
| seasonal | cmd_son | Hargreaves climatic moisture deficit in September, October and November |
| seasonal | dd5_djf | degree-days above 5°C in December, January and February |
| seasonal | dd5_jja | degree-days above 5°C in June, July and August |
| seasonal | dd5_mam | degree-days above 5°C in March, April and May |
| seasonal | dd5_son | degree-days above 5°C in September, October and November |
| annual | dd5 | degree-days above 5°C |
| seasonal | dd_0_djf | degree-days below 0°C in December, January and February |
| seasonal | dd_0_mam | degree-days below 0°C in March, April and May |
| seasonal | dd_0_son | degree-days below 0°C in September, October and November |
| annual | dd_0 | degree-days below 0°C |
| annual | emt | extreme minimum temperature over 30 years |
| annual | eref | Hargreaves reference evaporation |
| seasonal | eref_djf | Hargreaves reference evaporation in December, January and February |
| seasonal | eref_jja | Hargreaves reference evaporation in June, July and August |
| seasonal | eref_mam | Hargreaves reference evaporation in March, April and May |
| seasonal | eref_son | Hargreaves reference evaporation in September, October and November |
| annual | ext | extreme maximum temperature over 30 years |
| annual | map | mean annual precipitation (mm) |
| annual | mat | mean annual temperature (°C) |
| annual | mcmt | mean coldest month temperature (°C) |
| annual | mwmt | mean warmest month temperature (°C) |
| annual | nffd | the number of frost-free days |
| seasonal | nffd_mam | the number of frost-free days in March, April and May |
| seasonal | nffd_son | the number of frost-free days in September, October and November |
| annual | pas | precipitation as snow (mm) between August in previous year and July in current year |
| seasonal | pas_djf | precipitation as snow (mm) between August in previous year and July in current year in December, January and February |
| seasonal | pas_son | precipitation as snow (mm) between August in previous year and July in current year in September, October and November |
| seasonal | ppt_djf | precipitation in December, January and February |
| seasonal | ppt_jja | precipitation in June, July and August |
| seasonal | ppt_mam | precipitation in March, April and May |
| seasonal | ppt_son | precipitation in September, October and November |
| seasonal | tave_djf | mean temperature in December, January and February |
| seasonal | tave_jja | mean temperature in June, July and August |
| seasonal | tave_mam | mean temperature in March, April and May |
| seasonal | tave_son | mean temperature in September, October and November |
| annual | td | temperature difference between MWMT and MCMT, or continentality (°C) |
| seasonal | tmax_djf | maximum temperature in December, January and February |
| seasonal | tmax_jja | maximum temperature in June, July and August |
| seasonal | tmax_mam | maximum temperature in March, April and May |
| seasonal | tmax_son | maximum temperature in September, October and November |
| seasonal | tmin_djf | minimum temperature in December, January and February |
| seasonal | tmin_jja | minimum temperature in June, July and August |
| seasonal | tmin_mam | minimum temperature in March, April and May |
| seasonal | tmin_son | minimum temperature in September, October and November |

**Table S2.** Summary of GBS data.

| **Species** | **Cluster** | **Population** | **Location** | **Raw reads** | **Clean reads** | **Proportion** |
| --- | --- | --- | --- | --- | --- | --- |
| *T. koraiensis* |  | LYL | Laoyeling, Heilongjiang, China | 24,755,970 | 23,133,504 | 93.45% |
| *P. orientalis* | A | LY | Lingyuan, Liaoning, China | 133,659,084 | 124,600,368 | 93.22% |
| *P. orientalis* | A | YKZ | Yikezhao, Neimenggu, China | 23,485,634 | 21,959,987 | 93.50% |
| *P. orientalis* | A | HS | Heshui, Gansu, China | 15,605,472 | 14,391,793 | 92.22% |
| *P. orientalis* | A | HL | Huangling, Shaanxi, China | 14,562,490 | 13,570,556 | 93.19% |
| *P. orientalis* | A | PL | Pinglu, Shanxi, China | 6,787,158 | 6,329,073 | 93.25% |
| *P. orientalis* | A | LD | Liangdang, Gansu, China | 25,295,120 | 23,547,639 | 93.09% |
| *P. orientalis* | B | MY | Miyun, Beijing, China | 8,574,470 | 7,900,944 | 92.14% |
| *P. orientalis* | B | JC | Jiaocheng, Shanxi, China | 21,214,422 | 19,836,151 | 93.50% |
| *P. orientalis* | B | JCH | Jincheng, Shanxi, China | 10,480,588 | 9,820,746 | 93.70% |
| *P. orientalis* | B | CQ | Changqing, Shandong, China | 13,496,530 | 12,620,176 | 93.51% |
| *P. orientalis* | B | ZB | Zibo, Shandong, China | 25,254,886 | 23,465,612 | 92.92% |
| *P. orientalis* | B | HX | Huixian, Henan, China | 33,632,574 | 31,344,898 | 93.20% |
| *P. orientalis* | B | JX | Jiaxian, Henan, China | 5,474,624 | 5,083,436 | 92.85% |
| *P. orientalis* | C | QS | Queshan, Henan, China | 36,506,802 | 34,163,958 | 93.58% |
| *P. orientalis* | C | LN | Luonan, Shaanxi, China | 8,133,742 | 7,600,259 | 93.44% |
| *P. orientalis* | C | JS | Jishan, Shanxi, China | 36,853,540 | 34,444,835 | 93.46% |
| *P. orientalis* | C | NZ | Nanzheng, Shaanxi, China | 17,991,918 | 16,819,982 | 93.49% |
| *P. orientalis* | C | CL | Cili, Hunan, China | 5,169,926 | 4,828,058 | 93.39% |
| *P. orientalis* | C | LP | Liping, Guizhou, China | 10,358,832 | 9,556,328 | 92.25% |
| *P. orientalis* | C | NP | Nanping, Fujian, China | 9,126,844 | 8,481,099 | 92.92% |
| *P. orientalis* | Not defined | BT | Baotou, Neimenggu, China | 18,197,296 | 16,875,362 | 92.74% |

**Table S3.** Mean Weir and Cockerham’s (1984) *F*_ST_ among four clusters based on 3,911 unlinked SNPs and the full set of 11,049 SNPs (in parentheses).

|  | A | B | C |
| --- | --- | --- | --- |
| B | 0.030 (0.032) |  |  |
| C | 0.069 (0.068) | 0.034 (0.035) |  |
| *T.* *koraiensis* | 0.127 (0.125) | 0.105 (0.104) | 0.140 (0.137) |
